# Preoperative mechanical preparation of the colon: the patient's experience

**DOI:** 10.1186/1471-2482-7-5

**Published:** 2007-05-04

**Authors:** Barbel Jung, Olof Lannerstad, Lars Påhlman, Malin Arodell, Mitra Unosson, Erik Nilsson

**Affiliations:** 1 University of Umeå and Department of Surgery, Visby Hospital, Visby Sweden; 2 Department of Surgery, Kalmar Hospital, Kalmar Sweden; 3 Department of Surgery, Colorectal Unit, University Hospital, Uppsala, Sweden; 4 Department of Medicine and Care, Division of Nursing Science, Linköping University, Sweden; 5 Department of Surgery, University Hospital, Umeå, Sweden

## Abstract

**Background:**

Preoperative mechanical bowel preparation can be questioned as standard procedure in colon surgery, based on the result from several randomised trials.

**Methods:**

As part of a large multicenter trial, 105 patients planned for elective colon surgery for cancer, adenoma, or diverticulitis in three hospitals were asked to complete a questionnaire regarding perceived health including experience with bowel preparation. There were 39 questions, each having 3 – 10 answer alternatives, dealing with food intake, pain, discomfort, nausea/vomiting, gas distension, anxiety, tiredness, need of assistance with bowel preparation, and willingness to undergo the procedure again if necessary.

**Results:**

60 patients received mechanical bowel preparation (MBP) and 45 patients did not (No-MBP). In the MBP group 52% needed assistance with bowel preparation and 30% would consider undergoing the same preoperative procedure again. In the No-MBP group 65 % of the patients were positive to no bowel preparation. There was no significant difference between the two groups with respect to postoperative pain and nausea. On Day 4 (but not on Days 1 and 7 postoperatively) patients in the No-MBP group perceived more discomfort than patients in the MBP group, p = 0.02. Time to intake of fluid and solid food did not differ between the two groups. Bowel emptying occurred significantly earlier in the No-MBP group than in the MBP group, p = 0.03.

**Conclusion:**

Mechanical bowel preparation is distressing for the patient and associated with a prolonged time to first bowel emptying.

## Background

Mechanical bowel preparation (MBP) has until recently been thought to be one of the most important factors to decrease infectious complications and prevent anastomotic dehiscence after elective colorectal surgery, but its value has been questioned [1,2]. Several randomised studies [3-9] and meta-analyses [10-12] since the beginning of the 1990's have shown that omission of MBP does not increase the risk for anastomotic dehiscence or septic complications in colon surgery. In the largest, randomised multicenter trial performed we found that MBP did not lower the risk for cardiovascular, general infectious or surgical site complications [13].

The aim of this study was to compare the patients' experience and acceptance of preoperative MBP versus no preoperative MBP in elective colon surgery.

## Methods

The study population was recruited from patients participating in the Swedish Mechanical Bowel Preparation Study comparing the outcome after elective open colon surgery with or without mechanical bowel preparation [13]. The study used central randomization with facsimile transmission to the randomisation centre. The allocation result was returned the same day. Stratification was made for each participating unit and the patients were randomised in blocs of permutation of four using computer generated random numbers. The participating surgeons did not know the size of the blocs. The inclusion criteria were: elective surgery for cancer, adenoma or diverticular disease, age 18–85 years, ASA (American Society of Anesthesiologists) Classification I-III, and life expectancy 6 months or longer.

At three units participating in the trial, patients were asked to complete a questionnaire having 39 questions, each with 3–10 answer alternatives. The questionnaire was designed using parameters previously validated to describe preoperative and postoperative experience.

Patients completed questionnaires preoperatively and on Days 1, 4 and 7. The questions and answer alternatives are described in Table 1 (preoperative) and Table 2 (postoperative). Comparisons were made between patients receiving mechanical bowel preparation (MBP group) and patients without mechanical bowel preparation (No-MBP group) prior to surgery. The planned number to be included in the study was 100 patients. Data were recorded in a protocol and stored in an electronic database at the study centre in Motala, Sweden.

**Table 1:** The Patient's experience preoperatively.

**Question**	**Answer alternatives**
Describe your appetite the week before surgery?	Very good/Good/Fairly good/Fairly poor/Poor/Very poor
Did your diet change during the days prior to surgery?	Ate more than normal/Did not change/Ate less than normal
Were you able to complete your bowel preparation?	Yes, completely/to some extent
If you had difficulty in completing bowel preparation – which were the two most important reasons?	Free text
How would you describe your experience of your preoperative preparation?	0–10 on a Numerical Rating Scale where 0 = not difficult at all and 10 = extremely difficult
Did you experience pain from the abdomen/bowel the day before surgery?	0–10 on a Numerical Rating Scale where 0 = no pain and 10 = worst conceivable pain
Did you experience discomfort in the abdomen/bowel the day before surgery?	0–10 on a Numerical Rating Scale where 0 = no discomfort and 10 = worst conceivable discomfort
Did you experience hunger the day before surgery?	Not at all/A little/Quite a lot/Very much
Did you feel tired the day before surgery	Not at all/A little/Quite a lot/Much/Very much
Did you experience a sense of fullness the day before surgery?	Not at all/A little/Quite a lot/Very much
Did you experience nausea the day before surgery?	Not at all/A little/Quite a lot/Much/Very much
Did you experience abdominal distension the day before surgery?	Not at all/A little/Quite a lot/Much/Very much
Did you experience anxiety the day before surgery?	Not at all/A little/Quite a lot/Much/Very much
Did you feel sick the day before surgery?	Not at all/Quite a lot
Did you experience sleeping disturbance the day before surgery?	Not at all/A little/Quite a lot/Much/Very much
Did you experience disturbance in your daily routine the day before surgery?	Not at all/A little/Quite a lot/Much/Very much
Did you experience extensive sweating the day before surgery?	Not at all/A little/Quite a lot/Much
How many times did you visit the toilet the day before surgery?	None/1–2/3–4/5–6/7–8/9–10/10 or more
If you had bowel preparation, did you need assistance?	No assistance/Assistance from a relative/Assistance from hospital staff/assistance from other person
Could You consider the same preoperative preparation again?	Absolutely/Possibly/Absolutely not

**Table 2:** The Patient's experience postoperatively. Description of questionnaire

**Questions**	**Answer alternatives**
Describe the extent of pain you experienced during the first, fourth and seventh postoperative day.	A ten point Numerical Rating Scale where 0 = no pain and 10 = worst conceivable pain
How many times did you take pain medication during the seventh postoperative day?	Zero/Once/Twice/Three times/More than three times
Describe the extent of discomfort you experienced during the first, fourth and seventh postoperative day.	A ten point Numerical Rating Scale where 0 = no discomfort and 10 = worst conceivable discomfort
Describe the extent of nausea you experienced during the first, fourth and seventh postoperative day.	A ten point Numerical Rating Scale where 0 = no discomfort and 10 = worst conceivable discomfort
Do you experience constipation now on the seventh postoperative day?	Yes/No
Do you have diarrhoea now on the seventh postoperative day?	Yes/No
How would you describe your appetite now, on the seventh postoperative day?	Very good/Good/Fairly good/Fairly poor/Poor/Very poor
If you compare your appetite now with your appetite preoperatively, how would you describe it today on the seventh postoperative day?	Improved a lot/Improved/Improved to some extent/Not affected/Slightly worse/Worse/Very much worse
When did you drink for the first time postoperatively?	The day of surgery/The day after surgery/two days after surgery/More than two days after surgery
When did you have solid food for the first time postoperatively?	The day after surgery/two days/three days/four days/five days/six days/More than six days after surgery
When did you experience the movement of gas in the bowel postoperatively?	The day after surgery/two days after surgery/three days after surgery/four days after surgery/more than four days after surgery
When did you have your first bowel movement postoperatively?	The day after surgery/two days after surgery/three days after surgery/four days after surgery/more than four days after surgery
Where were you when you completed this form?	In the surgical department/in another hospital department/at home/with a relative

The Local Ethics Committee approved the study.

### Statistical analysis

The chi square test was used to test differences between categorical variables and the t-test or Mann-Whitney U-test for independent groups to test differences between continuous variables. Two tailed P-values < 0.05 were considered significant.

## Results

One hundred and five patients were included in the study between February 2000 and March 2002. Sixty patients received MBP and 45 had no MBP. The groups were balanced regarding age, gender, BMI and diagnosis as shown in Table 3. MBP was accomplished with sodium phosphate in 28 patients, polyethylene glycol in 31 patients, and enema in one case.

**Table 3:** Demographic data and diagnosis

	MBP N = 60	No-MBP N = 45	P-value
Age; years, mean	67.6	68.2	0.77
Male (%)	29 (48)	23 (51)	0.77
Diagnosis			0.67
Cancer (%)	46 (77)	31 (69)	
Adenoma (%)	5 (8)	5 (11)	
Diverticular disease (%)	9 (15)	9 (20)	
BMI mean (SD)	25.6 (3.7)	24.8 (4.0)	0.23

### Tolerability to bowel preparation

Four patients in the MBP group could not complete the intended MBP (two of these patients received sodium phosphate, and two polyethylene glycol). All four patients stated that the reason for not completing MBP was inability to drink the required amount of fluid. In addition, two patients mentioned nausea to be a problem.

Assistance with MBP (hospital staff or relative) was required by 30/58 (52%) of the patients in the MBP group. Sixteen per cent of patients in the MBP group reported more than 10 defecations the day before surgery.

### Questionnaire results

**Figure 1:**
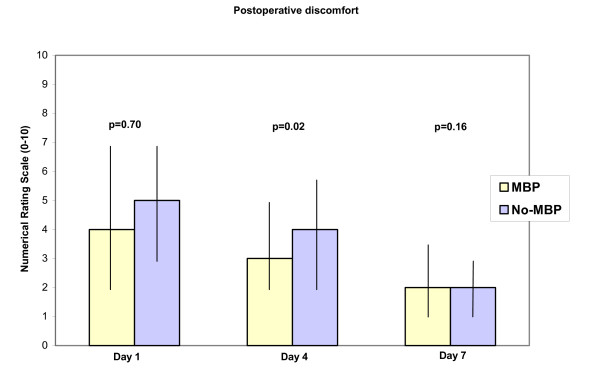
Height of boxes represents median of experienced discomfort on Days 1, 4 and 7 postoperatively, measured as value on a ten point Numerical Rating Scale. Vertical bars represent inter-quartile range. P < 0.05 considered significant (t-test).

**Figure 2:**
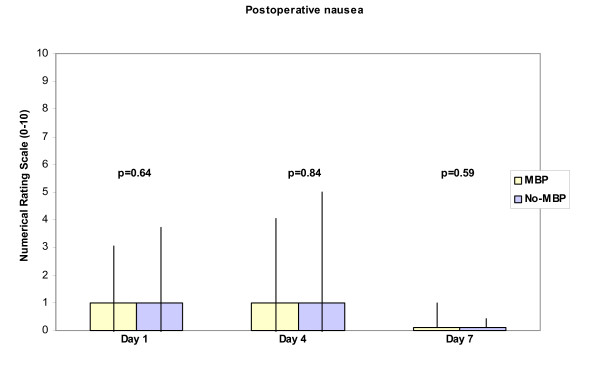
Height of boxes represents median of experienced nausea on Days 1, 4 and 7 postoperatively measured as value on a ten point Numerical Rating Scale. Vertical bars represent inter quartile-range. P < 0.05 considered significant (t-test).

**Figure 3:**
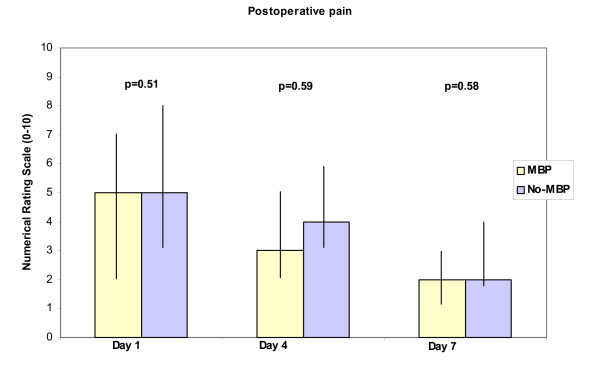
Height of boxes represents median of experienced pain on Days 1, 4 and 7 postoperatively measured as value on a ten point Numerical Rating Scale. Vertical bars represent inter-quartile range. P < 0.05 considered significant (t-test).

Only three statistically significant differences were noted between the two groups (Table 1, 2): willingness to consider the same preoperative procedure again time to first bowel movement (p = 0.04) and degree of discomfort on day 4 postoperatively (p = 0.02). The response rate in the No-MBP group to the question about the willingness to consider the same preoperative procedure again was low (58%) compared to the response rate in the MBP group (95%) (P < 0.001), see Table 4. Patients in the No-MBP group had their first bowel movement earlier than patients in the MBP group, see Table 5. Patients in the No-MBP group experienced a higher degree of discomfort (but not pain or nausea) on the fourth postoperative day compared to patients in the MBP group. For details see Figures 1,2,3.

**Table 4:** Could you consider the same preoperative preparation again?

	MBP N = 60	No-MBP N = 45
Absolutely	17 (28 %)	17 (38 %)
Possibly	32 (53 %)	8 (18 %)
Absolutely not	8 (13 %)	1 (2 %)
No answer	3 (5 %)	19 (42 %)

**Table 5:** Time to first bowel movement

	MBP (n = 56)	No-MBP (n = 41)
The day after surgery	2 (3.6%)	0
Two days after surgery	7 (12.5%)	4 (9.8%)
Three days after surgery	9 (16.1%)	13 (31.7%)
Four days after surgery	9 (16.1%)	13 (31.7%)
More than four days after surgery	29 (51.8%)	11 (26.8%)

P = 0.03 Fisher's exact test

## Discussion

In this study we have shown that patients prefer not to have MBP, that the time to first postoperative bowel movement is shorter without MBP, and that patients not receiving MBP experienced more discomfort on the fourth postoperative day.

The response rate to the question concerning willingness to consider the same preoperative procedure again was significantly lower in the No-MBP group compared to the MBP group, 58 Vs 95%. This most likely reflects the fact that many patients without MBP thought (correctly) that they did not receive any "preoperative procedure". However, for patients answering this question those in the MBP group were less inclined to consider the same preoperative procedure again (30% vs. 65%).

Different methods for MBP exist, of which whole bowel irrigation with oral polyethylene glycol and sodium phosphate solutions are most commonly used according to recent studies [3,4,8,14]. It has been shown that the patient acceptance is greater for sodium phosphate than for polyethylene glycol [15]. However, sodium phosphate is contraindicated in patients with renal or congestive heart failure due to its influence on electrolyte- and water balance which limits its use in many elderly patients.

Patients in the No-MBP group experienced a higher degree of abdominal discomfort on the fourth postoperative day than patients in the MBP group, but without any corresponding difference in perceived pain. One possible explanation for the inter-group difference is an earlier onset of bowel motility in the No-MBP group. The majority of patients in the No-MBP group had their first bowel movement on the third or fourth postoperative day compared to more than four days in the MBP group. Early bowel movement is compatible with accelerated rehabilitation and is thus an advantage [16].

The questionnaire used was designed for this study and was not validated prior to this study. The questions addressed to the patients were however chosen based on previous research [17-21].

Besides safety and patient preference there are economic issues to be addressed when deciding whether or not to change routines in preoperative bowel preparation. Our study showed that 52% of patients receiving MBP needed help from hospital staff or a relative. For many patients this requires admission to the surgical ward early the day before surgery, thereby increasing the workload on the personnel and the cost to society.

## Conclusion

It has previously been shown that mechanical bowel preparation does not reduce the rate of complications in colon surgery. This report demonstrates that mechanical bowel preparation is considered unpleasant by many patients and that it delays the return of normal bowel movements. Both these findings increase the incentive to omit mechanical bowel preparation in elective colon surgery.

## Competing interests

The authors declare that they have no competing interests.

## Authors' contributions

BJ participated in the design of the study, carried out the data collection and coordination of the study and drafted the manuscript. OL and LP participated in the design of the study and collection of data. MA designed the questionnaire. MU supervised the design of the questionnaire and participated in the design of the study. EN participated in the design of the study. All authors participated in the revision of the manuscript and approved the final manuscript.

## Pre-publication history

The pre-publication history for this paper can be accessed here:


